# Sperm midpiece apoptotic markers: impact on fertilizing potential in in vitro fertilization and intracytoplasmic sperm injection

**DOI:** 10.1007/s13577-015-0129-z

**Published:** 2016-01-20

**Authors:** Joanna Talarczyk-Desole, Małgorzata Kotwicka, Magdalena Jendraszak, Leszek Pawelczyk, Marek Murawski, Piotr Jędrzejczak

**Affiliations:** Division of Infertility and Reproductive Endocrinology, Poznan University of Medical Sciences, Polna 33, 60-535 Poznan, Poland; Department of Cell Biology, Poznan University of Medical Sciences, Rokietnicka 5d, 60-806 Poznan, Poland; 1st Department and Clinic of Gynaecology and Obstetrics, Wrocław Medical University, T. Chałubińskiego 3, 50-368 Wrocław, Poland

**Keywords:** Male infertility, IVF, ICSI, Spermatozoa, Phosphatidylserine translocation, Caspase-3, Apoptotic markers

## Abstract

The aim of this study was to investigate the relationship between apoptotic markers present in human spermatozoa, namely phosphatidylserine translocation (PST) from the inner to the outer layer of the cytomembrane and the active form of caspase-3 (c3) versus the fertilizing potential of male gametes in conventional in vitro fertilization (IVF) and intracytoplasmic sperm injection (ICSI) models. A total of 116 male patients treated with their partners for infertility underwent basic semen analysis and an assessment of the presence of PST and the active c3 in sperm using flow cytometry. Forty patients underwent IVF, group A, while 76 patients underwent ICSI, group B. The fertilizing potential of the gametes was measured as the percentage of oocytes with pronuclei present after either procedure. PST and active c3 were identified in vital gametes, mainly in the midpiece area. Concentration, motility, morphology, and viability of spermatozoa strongly negatively correlated with both markers. In group A, a negative correlation between both markers and the success rate of conventional IVF was observed (*r* = −0.4, *p* = 0.04 for PST; *r* = −0.4, *p* = 0.02 for active c3, respectively). In group B, the success rate of ICSI did not correlate with either marker (*r* = −0.2, *p* = 0.85 for PST and *r* = 0.1, *p* = 0.51 for active c3). The two apoptotic markers localized in the sperm midpiece area may affect their function not only by decreasing basic andrologic parameters but also by reducing the probability of conception. Therefore, analysis of PST and active c3 in the sperm of patients undergoing infertility treatment should be recommended.

## Introduction

Although in vitro fertilization (IVF) is the most commonly used and most efficient method of male infertility treatment, its outcome is still not satisfactory [1]. The quality of the sperm is one of the strongest elements influencing the success rate of the therapy [2]. Conventional sperm analysis is based on microscopic assessment of the concentration, motility, morphology, and vitality of spermatozoa according to World Health Organization (WHO) recommendations [3]. However, this analysis may not be sufficient because sperm parameters do not reflect the potential of the male gamete to penetrate the oocyte [4]. Recently, much attention has focused on the influence of apoptosis on sperm quality.

Apoptosis is the process of programmed cell death in multicellular organisms [5]. It has been suggested to regulate cell homeostasis during spermatogenesis by adjusting the number of germ cells relative to the number of Sertoli cells in testis [6]. In somatic cells, the apoptosis was described with typical cell shrinkage, chromatin condensation and fragmentation, cell vacuolization, and apoptotic bodies forming [7]. Spermatozoa cannot reveal such morphological signs of apoptosis due to particularity of their structure. Although the exact mechanism of apoptotic changes in spermatozoa is still unknown, some of the somatic cell markers typical for apoptosis are present in mature male gametes. Among other manifestations, apoptotic changes in sperm cells are demonstrated by impairment of cell membrane integrity. This phenomenon is evidenced by (1) the externalization of phosphatidylserine (PS) from the inner to the outer layer of the cytomembrane (early marker of apoptosis) [8] and (2) the presence of the active form of caspase-3 (c3), the apoptosis executor, in apoptotic gametes (late marker of apoptosis) [9]. Parallel, both phosphatidylserine translocation (PST) and c3 were observed in cells not being involved in apoptosis; for example, PST is related to exo- and endocytosis [10] or myotube formation [11]. Some studies show that PS externalization may also occur following capacitation [12, 13]. On the other hand, the c3 together with other caspases participates in cell differentiation and proliferation as well as in receptor internalizations [14–16].

Spermatozoa presenting PST or active c3 are often described as apoptotic. However, such terminology can be misleading. Although both PS externalization and caspase activation may participate in elimination of defected spermatozoa, the definition of apoptosis seems not to be fulfilled in this case. Thus, in this study, these spermatozoa will be described as having apoptotic markers or apoptotic signaling.

The results regarding the correlation between apoptotic signaling and sperm parameters are conflicting. Higher PST was found in men with lower sperm quality than sperm donors [17–19]. The presence of PST is related to lower vitality and motility of the sperm cells [20]. In contrast, Ricci et al. [21] did not find correlation between PST and sperm parameters [21]. Regarding the active form of c3, Weng et al. [17] showed its association with poorer concentration, morphology, and progressive motility of the spermatozoa. Few studies have been performed to evaluate the influence of apoptotic signaling on the fertilizing potential of the male gamete. Grunewald et al. [22] found decreased penetration of zona-free hamster oocytes by spermatozoa with PST, while Dirican et al. [23] found an increased fertilization rate in intracytoplasmic sperm injection (ICSI) after using magnetic cell sorting (MACS) to eliminate male gametes with PST. Marchetti et al. [24] found a negative correlation between the presence of the active form of c3 and conventional IVF outcome. Therefore, it could be hypothesized that apoptotic signaling may impair one of the most important functions of the male gamete, namely the fertilizing potential.

The aim of the present study was to investigate the correlation between apoptotic markers in spermatozoa and the fertilizing potential of male gametes using conventional IVF and ICSI models. The evaluation of the influence of apoptotic signaling on the outcome of these two methods of IVF may elucidate the fertilization stage in which apoptotic processes play the most important role and suggest how apoptotic signaling is related to the function of the male gamete.

### Ethics statement

This study was performed with the approval of the Bioethical Committee, Poznan University of Medical Sciences, Number 425/11. All participants were informed of the aim of the study and signed a written agreement to participate in it.

## Methods

### Materials

The study group consisted of 116 men of European descent who were treated with their partners for infertility from January 2011 to November 2012. All couples participated in the IVF program. Idiopathic infertility, tubal factors, and male factors of infertility were inclusion criteria of the assessment. Exclusion criteria for females consisted of age over 39 years, FSH in the 3rd–5th day of the cycle over 12 mIU/mL, grade III or IV endometriosis according to the American Society of Reproductive Medicine (ASRM), and polycystic ovary syndrome. Exclusion criteria for males consisted of hypogonadotropic hypogonadism and azoospermia.

All 116 couples underwent IVF procedures. The seminal parameters assessed on the day of oocyte retrieval were used to determine eligibility for either conventional IVF or ICSI. The patients who underwent conventional IVF were included in group A (40 couples), and the patients who underwent ICSI were included in group B (76 couples). The fertilizing potential of the gametes was measured by the percentage of oocytes with pronuclei present after either IVF procedure. Therefore, only couples with at least two oocytes per IVF procedure (conventional IVF or ICSI) were qualified for the study. The sperm of all patients were analyzed in two stages. First, before oocyte retrieval from the female partners, the ejaculates underwent standard seminal analysis. Then, after the IVF procedure, the remnants of the sperm samples of 85 patients were examined for markers of apoptosis. The remnant was the volume of sperm sample of each patient, which was left after IVF procedure. Until the markers of apoptosis were analyzed, the remnants were carefully stored in temperature of 37 °C. The time between beginning of either IVF procedure or flow cytometry was never longer than 1 h, so the quality of sperm in both procedures was comparable. All samples were analyzed for phosphatidylserine membrane translocation, and 69 samples were examined for the presence of the active form of caspase-3. An analysis of the other 16 samples for active c3 could not be performed due to insufficient volume of the ejaculate remnants.

### Seminal analysis

Semen samples were obtained after 2–5 days of sexual abstinence on the day of ovarian puncture. The ejaculates were processed and examined according to WHO guidelines (2010), and parameters such as the concentration, motility, progressive motility, morphology, and viability (by means of a hypo-osmotic swelling test) of each sample were assessed [3].

### Phosphatidylserine membrane translocation

To determine PST from the inner to the outer layer of the plasma membrane, annexin-V labeled with fluorescein (AnV-FITC) (Molecular Diagnostics, Darmstadt, Germany) was used. Simultaneously, to distinguish between viable and dead spermatozoa, propidium iodide (PI) staining at a final concentration of 0.125 μg/L (Sigma-Aldrich, St. Louis, MO) was used. Double staining was conducted according to the manufacturer’s recommendations. The analysis was performed with a confocal microscope (LSM 510, Zeiss, Germany) and flow cytometer (FACS Calibur, Becton–Dickinson, USA).

### Active caspase-3

The caspase-3 inhibitor DEVD-FMK conjugated with fluorescein (FITC-DEVD-FMK) (Calbiochem, Darmstadt, Germany) was used to detect spermatozoa with active caspase-3. FITC-DEVD-FMK is cell-permeable and nontoxic, and it irreversibly binds to activated caspase-3 in apoptotic cells. The gametes were incubated with FITC-DEVD-FMK for 45 min at 37 °C in 5 % CO_2_, according to the manufacturer’s instructions. After 35 min of incubation, 1 μL of PI (50 μL/mL) was added, and the suspension was further incubated for 10 min to differentiate between vital and necrotic cells. Subsequently, the cells were washed twice with washing buffer, and the fluorescence intensity was then measured using a flow cytometer. The staining was also analyzed with a confocal microscope.

### Flow cytometry

The fluorescence signals of spermatozoa with PST and active caspase-3 were measured by a FACS Calibur flow cytometer (Becton–Dickinson, USA) to determine their viability. A total of 10,000 cells were analyzed in each experiment. The green fluorescence of An-V-FITC and active caspase-3 was excited by an argon laser (488 nm) and detected on the FL1 channel (515–545 nm). The red fluorescence of PI was also excited by an argon laser (488 nm) and detected on the FL3 channel (>650 nm). CellQuest Pro software (v.5.2.1) (Becton–Dickinson) was used for the data analysis.

### In vitro fertilization procedure

All patients underwent controlled ovarian hyperstimulation to achieve an appropriate number of oocytes. The patients’ responses were monitored by transvaginal ultrasonography (TVS), and the level of 17β-estradiol was detected. A long protocol using a GnRH agonist based on LH suppression was performed by administrating a GnRH analog (0.1 mg/day Triptorelin, Gonapeptyl Daily—Ferring) on the twenty-first day of the cycle. On the third day of the cycle, after confirming the pituitary desensibilization criteria (follicle diameter <5 mm, endometrium thickness <4 mm, 17β-estradiol level <40 pg/mL), recombined gonadotropin (Gonal F, Serono, 150–225 IU sc/day) was administered. When the dominant follicles reached dimensions of at least 18 mm and a serum estradiol level of 150–200 pg/mL for each follicle, 10,000 IU of human chorionic gonadotropin (HCG) (Pregnyl, Organon) was administered intramuscularly to trigger final oocyte maturation. The follicle puncture was performed through vaginal fornices to obtain follicular fluid and oocytes 36 h after HCG administration under TVS supervision. The oocytes were collected, warmed in G-MOPS Plus medium (Vitrolife, Sweden), and placed in a CO_2_ incubator (Heracell, Haereaus, Austria). Only mature oocytes (in stage II metaphase of meiosis; MII) were used for the IVF procedure.

After liquefaction and standard andrologic assessment (WHO, 2010), the ejaculate was selected on a Sil-Select gradient (Ferti Pro, Belgium) according to the manufacturer’s instructions [25].

For the conventional IVF procedure, oocytes were placed in 4-well dishes with G-FERT Plus medium (Vitrolife, Sweden). Three hours after oocyte retrieval, 100,000–150,000 motile sperm were inseminated in each oocyte.

For ICSI, the mature oocytes were placed on 4-well dishes with G-FERT Plus medium (Vitrolife, Sweden) and covered with a mineral oil layer (OVOIL, Vitrolife, Sweden). Selected spermatozoa of normal motility and morphology were immobilized. Single sperm injection was performed by puncture of the zona pellucida and oolemma with a micropipette. After the procedure, the cells were placed in G-1 medium (Vitrolife, Sweden), covered with mineral oil (OVOIL, Vitrolife, Sweden), and placed in a CO_2_ incubator (Heracell, Haereaus, Austria).

In all IVF procedures, 16–18 h after insemination, oocytes were examined for the presence of pronuclei and polar bodies. Fertilization was considered normal when two pronuclei per oocyte were observed.

### Statistical analysis

Normal distribution of the data was demonstrated by the Kolmogorov–Smirnov test. The Mann–Whitney *U* test was used to compare sperm parameters between the conventional IVF and ICSI groups. Spearman’s correlation was calculated between the sperm parameters and apoptosis and between apoptosis and the fertilization outcome. Statistical analysis was performed using Statistica 10.0 software (StatSoft, Tulsa, OK, USA). A *p* value of <0.05 was considered statistically significant.

## Results

### Seminal parameters and their correlation with sperm apoptotic markers

The seminal parameters of patients in groups (A) and (B) are presented in Table 1. The concentration, motility, morphology, and viability of spermatozoa were significantly better in group A, but no statistically significant difference in apoptotic signaling was observed between the groups (*p* = 0.2 for spermatozoa with PST and *p* = 0.1 for spermatozoa with active c3).

**Table 1:** Mean value of sperm parameters and apoptosis markers in patients with conventional IVF and ICSI

Sperm parameters	Group A—conventional IVF		Group B—ICSI		*p* value
	Mean ± SD	*n*	Mean ± SD	*n*	
Concentration (mln/mL)	39 ± 16	40	18 ± 18	76	<0.0001
Progressive motility (%)	25 ± 90	40	14 ± 10	76	<0.0001
Total motility (%)	47 ± 12	40	32 ± 18	76	<0.0001
Morphology (%)	5 ± 20	40	3 ± 20	76	<0.0001
HOS (%)	62 ± 11	40	48 ± 19	76	<0.0001
Sperm with PST (%)	5 ± 40	28	9 ± 12	57	0.2
Sperm with active c3 (%)	21 ± 20	27	28 ± 25	42	0.1

*SD* standard deviation, *HOS* hypo-osmotic swelling test, *PST* phosphatidylserine membrane translocation, *c3* caspase-3

**Fig. 1:**
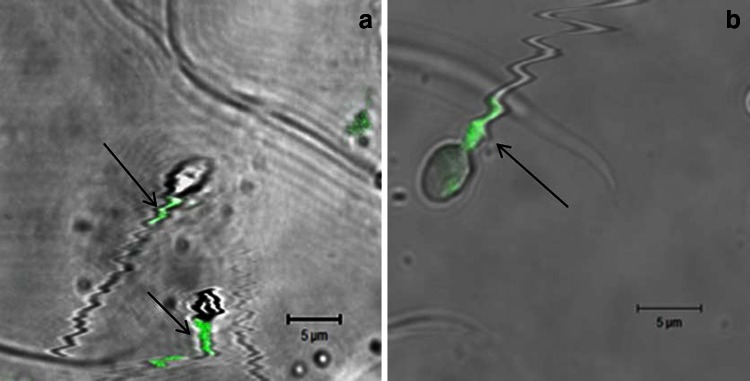
Photograph of vital spermatozoa exhibiting apoptotic signaling in the midpiece area (confocal microscope, magnification ×63). **a** Spermatozoa with phosphatidylserine translocation (annexin-V labeled with fluorescein staining). **b** Spermatozoon with the active form of caspase-3 (caspase-3 inhibitor labeled with fluorescein staining)

Microscopic assessment with a confocal microscope showed apoptotic signaling mainly in the midpiece area of the sperm for both AnV (Fig. 1a) and c3 (Fig. 1b) stainings. Flow cytometry analysis revealed the presence of both annexin-V-positive (AnV^+^) and active caspase-3-positive (c3^+^) fractions, which are considered cellular markers of apoptosis. Apoptotic signaling in vital gametes was visible mainly in the midpiece area.

Sperm parameters were tested for correlation with apoptotic signaling. In all patients, the concentration, motility, morphology, and viability were strongly negatively correlated with both markers of apoptosis. The presence of PST was positively correlated with the presence of the active form of c3 (*r* = 0.6, *p* < 0.001; Table 2).

**Table 2:** Correlation between sperm parameters and apoptotic signaling—Spearman rank correlation coefficient (*r*)

	Sperm with PST	Sperm with active c3	Concentration	Progressive motility	Motility	Morphology	HOS
Sperm with PST	–	0.6**	−0.4*	−0.4**	−0.4*	−0.3*	−0.3*
Sperm with active c3	0.6**	–	−0.5**	−0.6**	−0.5**	−0.4*	−0.5**

*PST* phosphatidylserine membrane translocation, *c3* caspase-3, *HOS* hypo-osmotic swelling test

* *p* < 0.001; ** *p* < 0.0001

### Correlation of sperm apoptotic markers with the fertilizing potential of male gametes

**Fig. 2:**
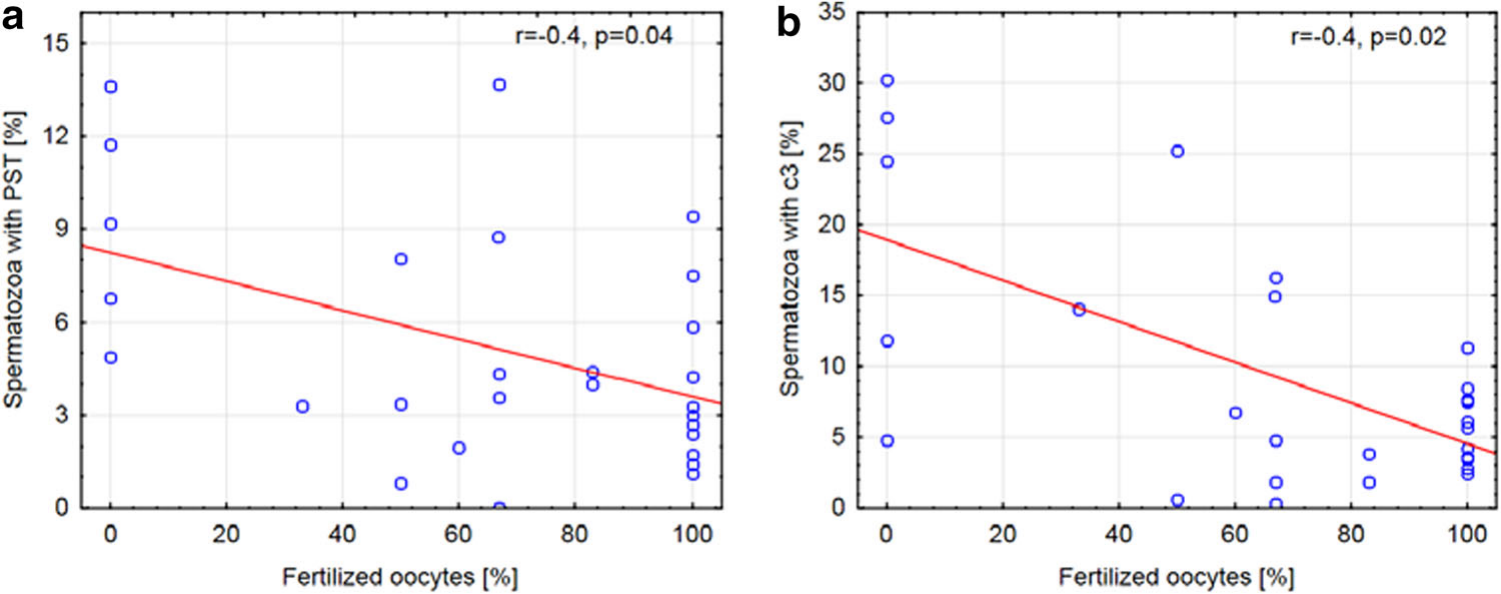
Correlation between the fertilization rate in conventional IVF and markers of apoptosis. **a** Correlation between spermatozoa with PST and the percentage of oocytes fertilized using conventional IVF. **b** Correlation between spermatozoa with the active form of c3 and the percentage of oocytes fertilized using conventional IVF. *PST* phosphatidylserine membrane translocation, *c3* caspase-3, *IVF* in vitro fertilization

**Fig. 3:**
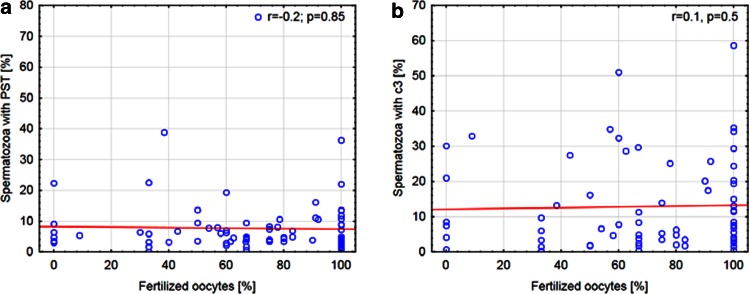
Correlation between the fertilization rate in ICSI and markers of apoptosis. **a** Correlation between spermatozoa with PST and the percentage of oocytes fertilized using ICSI. **b** Correlation between spermatozoa with the active form of c3 and the percentage of oocytes fertilized using ICSI. *PST* phosphatidylserine membrane translocation, *c3* caspase-3, *ICSI* intracytoplasmic sperm injection

The relationship between markers of apoptosis and the percentage of oocytes fertilized for both methods of IVF was analyzed. In group A, a negative correlation between spermatozoa with PST and the success rate of conventional IVF was observed (*r* = −0.4, *p* = 0.04). Additionally, the presence of the active form of c3 in sperm negatively correlated with the percentage of fertilized oocytes in this procedure (*r* = −0.4, *p* = 0.02; Fig. 2). In group B, the success rate of ICSI did not correlated with markers of apoptosis (*r* = −0.2, *p* = 0.85 and *r* = 0.1, *p* = 0.51 for An-V^+^ and c3^+^ spermatozoa, respectively, Fig. 3).

**Fig. 4:**
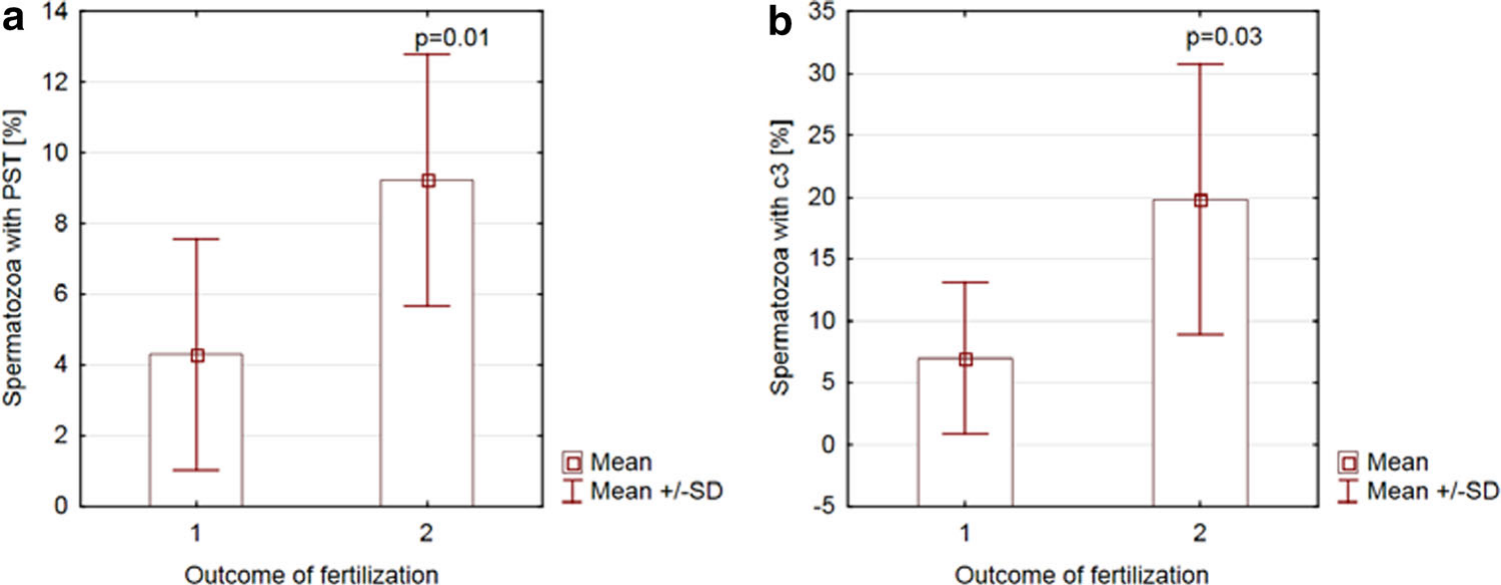
Comparison of apoptosis signaling between groups that accomplished (*1*) or did not accomplish (*2*) fertilization of at least one oocyte for conventional IVF. **a** Spermatozoa with PST in groups that accomplished (*1*) or did not accomplish (*2*) fertilization of at least one oocyte in conventional IVF. **b** Spermatozoa with the active form of c3 in groups that accomplished (*1*) or did not accomplish (*2*) fertilization of at least one oocyte in conventional IVF. *PST* phosphatidylserine membrane translocation, *c3* caspase-3, *IVF* in vitro fertilization

**Fig. 5:**
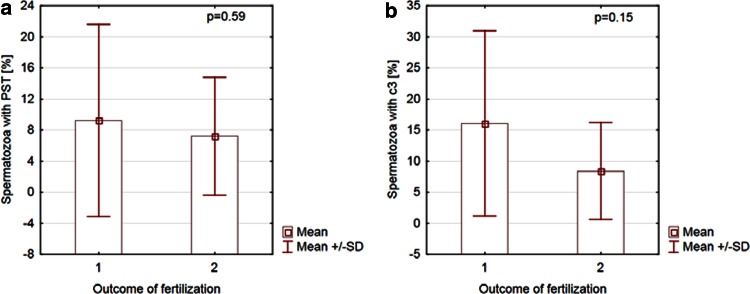
Comparison of apoptosis signaling between groups that accomplished (*1*) or did not accomplish (*2*) fertilization of at least one oocyte for ICSI. **a** Spermatozoa with PST in groups that accomplished (*1*) or did not accomplish (*2*) fertilization of at least one oocyte for ICSI. **b** Spermatozoa with the active form of c3 in groups that accomplished (*1*) or did not accomplish (*2*) fertilization of at least one oocyte for ICSI; *PST* phosphatidylserine membrane translocation, *c3* caspase-3, *ICSI* intracytoplasmic sperm injection

Next, all of the patients were divided into two groups: group (1), where at least one oocyte was fertilized, and group (2), where fertilization was not completed in any oocyte. A comparison of apoptotic signaling between group (1) and group (2) was performed for both IVF methods. Among the patients who underwent conventional IVF, the number of cells with both markers of apoptosis was significantly higher in group (2) than in group (1), *p* = 0.01 for gametes with PST and *p* = 0.03 for sperm with the active form of c3 (Fig. 4). However, no difference in apoptosis signaling between groups (1) and (2) was found for ICSI (*p* > 0.05 for both markers of apoptosis; Fig. 5).

## Discussion

Although spermatozoa parameters are routinely assessed according to the WHO guidelines, numerous studies suggest that spermatozoa function is associated with more than just their concentration, motility, morphology, and vitality. This is why the predictive value of these parameters for estimating fertilization success is questionable [4, 26, 27]. The methods used in this study allowed for a more complex evaluation of spermatozoa function. Standard assessment of the ejaculate according to the WHO guidelines was performed to select patients for either of the IVF procedures. Conventional IVF may be considered a model of natural conception, in which during all stages of fertilization, the spermatozoon has to function properly in order to fertilize the oocyte. In the ICSI model, the stages of recognition, fusion, and penetration of the oolemma are followed. A comparison of outcomes of both IVF methods can suggest which stages of fertilization may be affected by certain factors or processes. Assessing the fertilizing potential of spermatozoa at the early stage based on the presence of two pronuclei in the mature oocyte eliminates many factors that can influence the process of further embryo development and mirrors the role of male gametes in fertilization. Finally, testing the spermatozoa for apoptotic signaling provides an opportunity to assess the association of apoptosis-like processes in spermatozoa and sperm parameters with the outcome of IVF. An evaluation of the correlation of apoptotic signaling and both IVF methods has not previously been presented.

Average sperm parameters in our study group were close to normal values according to the WHO manual and significantly better in patients chosen for conventional IVF (group A) than in patients selected for ICSI (group B). However, there was no significant difference between groups A and B with respect to apoptotic signaling. Interestingly, this lack of a difference between groups was observed for both the early apoptotic marker (PST) and the marker of an apoptosis executor (the active form of c3). The similar levels of apoptotic markers found in groups fertilized with both IVF methods is an important feature of our study because it provides the opportunity to objectively compare the influence of apoptotic markers on the fertilization rate between the two groups.

Microscopic assessment of the samples apart from examination by flow cytometry allowed us to localize the position of apoptotic signaling in the gametes. Similar to our previous study, fluorescence was found mainly in the midpiece of the sperm and was also present in vital, motile spermatozoa [8]. The midpiece is the part of the spermatozoon where most of the organelles are localized. The dominant organelle of the sperm is the mitochondrion, which provides energy not only for cell movement but also for triggering the fertilization process. The presence of apoptosis in the midpiece area can possibly impair the function of mitochondria and, therefore, decrease the fertilizing potential of spermatozoa by decreasing the power of male gamete to cross the border of oolemma. This could explain the negative correlation of AnV^+^ and c3^+^ sperm with the outcome of conventional IVF, while the ICSI outcome was unaffected. Because apoptotic markers are also present in cells described as vital and motile according to the WHO criteria, impairment of cell function can be observed in standard ejaculate analysis and, therefore, affects the results of IVF.

In our study, the correlation between two apoptotic markers and sperm parameters was very strong, in contrast to the findings of Ricci et al. [21]. This result may be due to inclusion of fewer patients in their study. However, after selecting patients with normozoospermia from the total number of samples, they also found a negative correlation between apoptotic markers and sperm concentration.

The relationship between the apoptosis-like process and the fertilizing potential of spermatozoa was tested by various methods. We examined general tendencies by dividing the couples into positive and negative outcome groups, and we performed a more detailed analysis by correlating the percentage of fertilized oocytes with the examined apoptotic markers. Both methods confirmed a negative correlation of annexin-positive cells and cells with the active form of caspase-3 with the outcome of conventional IVF. However, neither parameter showed an association with the fertilization rate of ICSI. This result was also obtained in group A alone, where not only conventional IVF but also ICSI was performed (results not published). The oocytes were processed in both IVF methods with spermatozoa from the same ejaculate sample, and apoptotic signaling also showed no correlation with the outcome of ICSI (*r* = −0.2, *p* = 0.28 and *r* = −0.1, *p* = 0.72 for An-V^+^ and active c3^+^ spermatozoa, respectively). A decreased success rate of conventional IVF in patients with high apoptotic signaling showed that the externalization of phosphatidylserine and the presence of the active form of caspase-3 can disturb the processes of oocyte recognition, fusion with the oolemma, and penetration of the sperm. This is supported by Geunewald et al. [22] who reported that apoptosis-related signaling appeared to have negative association with sperm–oocyte penetration. These authors demonstrated strong negative correlation between activation of caspase-3 as well as PS externalization and the performance of the spermatozoa in the hamster oocyte penetration assay (SPA). Previous clinical studies showed that the result of the SPA correlated with the outcome of human IVF [28]. In such a context, our observation about positive correlation between sperm apoptotic markers and the fertilization rate of conventional IVF appears well grounded. The midpiece location of the PST and activation of caspase-3 suggest mitochondrial dysfunction, both by the location itself and by decreased sperm motility [20] resulting in impaired fertility potential. Nevertheless, the active caspase-3 presence does not represent a decisive indicator for cell death as the time of spermatozoa vital time was showed not to increase after incubation with caspases inhibitors [29].

In contrast to conventional IVF, fertilization rate in ICSI was unaffected by increased apoptotic markers. Presuming that sperm apoptotic markers decrease the sperm–oocyte penetration, the stage non-existing in the ICSI procedure, our results find a logical interpretation. These results were in accordance with the studies by Paasch et al. [31] and Grunewald et al. [22, 30], in which a negative influence of apoptosis on penetration and capacitation was suggested in a hamster oocyte model. However, because the percentage of oocytes fertilized in ICSI remains unaffected by the apoptosis-like process in spermatozoa, the initiation of nuclear decondensation may not be disturbed either, which, along with the presence of apoptotic markers mainly in the midpiece area of spermatozoon, supports the theory that increased apoptosis signaling of the sperm decreases fertilizing potential of male gamete primarily by diminishing penetrative capacity of it. Moreover, considering the midpiece located apoptotic markers to be also the mitochondrial dysfunction markers and the fact that in the final stage of fertilization the mitochondria are not transferred into the oocyte, it may explain the lack of correlation between the sperm midpiece apoptotic markers and the fertilization rate in ICSI procedure, as observed in this study.

## Conclusions

Our findings show that the apoptotic markers in spermatozoa may seriously affect their function not only by decreasing basic andrologic parameters but also by reducing the probability of conception. A high percentage of apoptosis-related cells may negatively influence the fertilization process in conventional IVF and also in natural conditions. Therefore, an analysis of apoptotic markers in the sperm of patients undergoing infertility treatment should be recommended. Sperm selection, for example, by MACS, to reduce the number of apoptotic-like cells in the samples used for IVF, can be a useful procedure to improve the outcome of assisted reproductive techniques.

## Abbreviations

ASRM: American Society of Reproductive Medicine
An-V: Annexin-V
c3: Caspase-3
FSH: Follicle-stimulating hormone
GnRH: Gonadotropin-releasing hormone
HCG: Human chorionic gonadotropin
ICSI: Intracytoplasmatic sperm injection
IVF: In vitro fertilization
LH: Luteinizing hormone
MACS: Magnetic-activated cell sorting
PST: Phosphatidylserine translocation
TVS: Transvaginal sonography
WHO: World Health Organization

## References

[1] Sunderam S, Kissin DM, Crawford S (2013). Assisted reproductive technology surveillance—United States, 2010. MMWR Surveill Summ.

[2] Jedrzejczak P, Taszarek-Hauke G, Hauke J, Pawelczyk L, Duleba AJ (2008). Prediction of spontaneous conception based on semen parameters. Int J Androl.

[3] WHO (2010). World Health Organization reference values for human semen characteristics.

[4] Jedrzejczak P, Pawelczyk L, Taszarek-Hauke G, Kotwicka M, Warchol W, Kurpisz M (2005). Predictive value of selected sperm parameters for classical in vitro fertilization procedure of oocyte fertilization. Andrologia.

[5] Kerr JF, Wyllie AH, Currie AR (1972). Apoptosis: a basic biological phenomenon with wide-ranging implications in tissue kinetics. Br J Cancer.

[6] Aitken RJ, Findlay JK, Hutt KJ, Kerr JB (2011). Apoptosis in the germ line. Reproduction.

[7] Wyllie AH, Kerr JF, Currie AR (1980). Cell death: the significance of apoptosis. Int Rev Cytol.

[8] Kotwicka M, Jendraszak M, Jedrzejczak P (2011). Phosphatidylserine membrane translocation in human spermatozoa: topography in membrane domains and relation to cell vitality. J Membr Biol.

[9] Kotwicka M, Filipiak K, Jedrzejczak P, Warchol JB (2008). Caspase-3 activation and phosphatidylserine membrane translocation in human spermatozoa: is there a relationship?. Reprod Biomed Online.

[10] Ikeda M, Kihara A, Igarashi Y (2006). Lipid asymmetry of the eukaryotic plasma membrane: functions and related enzymes. Biol Biophys Res Commun.

[11] Van den Eijnde SM, van den Hoff MJ, Reutelingsperger CP (2001). Transient expression of phosphatidylserine at cell–cell contact areas in required for myotube formation. J Cell Sci.

[12] Grunewald HJ, Baumamm T, Paasch U (2006). Capacitation and acrosome reaction in nonapoptotic human spermatozoa. Ann NY Acad Sci.

[13] Salicioni AM, Platt MD, Wertheimer EV (2007). Signalling pathways involved in sperm capacitation. Soc Reprod Fertil Suppl.

[14] Garrido C, Kromer G (2004). Life’s smile, death’s grin: vital functions of apoptosis-executing proteins. Curr Opin Cell Biol.

[15] Kumar S (2007). Caspases and their many biological functions. Cell Death Differ.

[16] Schwerk C, Schulze-Osthoff K (2003). Non-apoptotic functions of caspases in cellular proliferation and differentiation. Biochem Pharmacol.

[17] Weng SL, Taylor SL, Morshedi M (2002). Caspase activity and apoptotic markers in ejaculated human sperm. Mol Hum Reprod.

[18] Barroso G, Morshedi M, Oehninger S (2000). Analysis of DNA fragmentation, plasma membrane translocation of phosphatidylserine and oxidative stress in human spermatozoa. Hum Reprod.

[19] Paasch U, Agarwal A, Gupta AK (2003). Apoptosis signal transduction and the maturity status of human spermatozoa. Ann NY Acad Sci.

[20] Kotwicka M, Jendraszak M, Skibinska I, Jedrzejczak P, Pawelczyk L (2013). Decreased motility of human spermatozoa presenting phosphatidylserine membrane translocation-cells selection with the swim-up technique. Hum Cell.

[21] Ricci G, Perticarari S, Fragonas E (2002). Apoptosis in human sperm: its correlation with semen quality and the presence of leukocytes. Hum Reprod.

[22] Grunewald S, Said TM, Paasch U, Glander HJ, Agarwal A (2008). Relationship between sperm apoptosis signalling and oocyte penetration capacity. Int J Androl.

[23] Dirican EK, Ozgun OD, Akarsu S (2008). Clinical outcome of magnetic activated cell sorting of non-apoptotic spermatozoa before density gradient centrifugation for assisted reproduction. J Assist Reprod Genet.

[24] Marchetti C, Gallego MA, Defossez A, Formstecher P, Marchetti P (2004). Staining of human sperm with fluorochrome-labeled inhibitor of caspases to detect activated caspases: correlation with apoptosis and sperm parameters. Hum Reprod.

[25] Depa-Martynow M, Jedrzejczak P, Pawelczyk L (2007). Pronuclear scoring as a predictor of embryo quality in in vitro fertilization program. Folia Histochem Cytobiol.

[26] Lazaros LA, Xita NV, Kaponis AI, Zikopoulos KA, Plachouras NI, Georgiou IA (2009). Estrogen receptor and polymorphisms are associated with semen quality. J Androl.

[27] Depa-Martynow M, Kempisty B, Jagodzinski PP, Pawelczyk L, Jedrzejczak P (2012). Impact of protamine transcripts and their proteins on the quality and fertilization ability of sperm and the development of preimplantation embryos. Reprod Biol.

[28] Shy KK, Stenchever MA, Muller CH (1988). Sperm penetration assay and subsequent pregnancy—a prospective-study of 74 infertile man. Obstet Gynecol.

[29] Weil M, Jacobson MD, Raff MC (1998). Are caspases involved in the death of cells with a transcriptionally inactive nucleus? Sperm and chicken erythrocytes. J Cell Sci.

[30] Grunewald S, Kriegel C, Baumann T, Glander HJ, Paasch U (2009). Interactions between apoptotic signal transduction and capacitation in human spermatozoa. Hum Reprod.

[31] Paasch U, Grunewald S, Glander HJ (2007). Sperm selection in assisted reproductive techniques. Soc Reprod Fertil Suppl.

